# *Tg*SWO from *Trichoderma guizhouense* NJAU4742 promotes growth in cucumber plants by modifying the root morphology and the cell wall architecture

**DOI:** 10.1186/s12934-019-1196-8

**Published:** 2019-09-03

**Authors:** Xiaohui Meng, Youzhi Miao, Qiumei Liu, Lei Ma, Kai Guo, Dongyang Liu, Wei Ran, Qirong Shen

**Affiliations:** 1 Jiangsu Provincial Key Lab of Solid Organic Waste Utilization, Jiangsu Collaborative Innovation Center of Solid Organic Wastes, Educational Ministry Engineering Center of Resource-Saving Fertilizers, Nanjing Agricultural University, Nanjing, 210095 Jiangsu People’s Republic of China; 2 Biology Institute, Qilu University of Technology (Shandong Academy of Sciences), Jinan, 250014 Shandong People’s Republic of China

**Keywords:** *Trichoderma guizhouense* NJAU4742, Cucumber, *Tg*SWO, Root colonization, Growth promotion, Root architecture

## Abstract

**Background:**

Colonization of *Trichoderma* spp. is essential for exerting their beneficial functions on the plant. However, the interactions between *Trichoderma* spp. and plant roots are still not completely understood. The aim of this study was to investigate how *Tg*SWO affect *Trichoderma guizhouense* to establish themselves in the plant rhizosphere and promote plant growth. In this study, we deeply analyzed the molecular mechanism by which the functional characterization of the *Tg*SWO by expressing different functional region deletion proteins (FRDP) of *Tg*SWO.

**Results:**

Root scanning analysis results showed that *Tg*SWO could dramatically increase root density and promote growth. In addition, we also found that *Tg*SWO could expand root cell walls, subsequently increase root colonization. Moreover, knockout of *Tg*SWO mutants (KO) or overexpression of *Tg*SWO mutants (OE) produced greatly reduced or increased the number of cucumber root, respectively. To clarify the molecular mechanism of *Tg*SWO in plant-growth-promotion, we analyzed the ability of different FRDP to expand the root cell wall. The root cell wall architecture were considerably altered when treated by ΔCBD protein (the *Tg*SWO gene of lacking in the CBD domain was cloned and heterologously expressed), in correlation with the present YoaJ domain of *Tg*SWO. In contrast, neither the expansion of cell walls nor the increase of roots was detectable in ΔYoaJ protein.

**Conclusions:**

Our results emphasize the YoaJ domain is the most critical functional area of *Tg*SWO during the alteration of cell wall architecture. Simultaneously, the results obtained in this study also indicate that *Tg*SWO might play a plant-growth-promotion role in the *Trichoderma*-plant interactions by targeting the root cell wall.
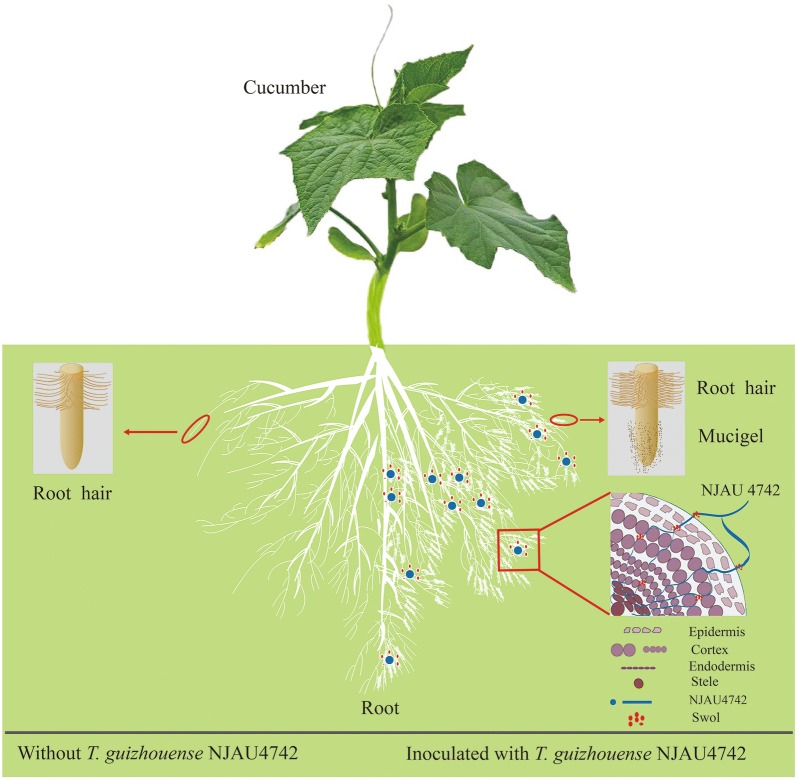

## Background

*Trichoderma* spp. are some of the most widely distributed plant growth-promoting microbes (PGPM) in agroecosystems worldwide [1, 2], and they exhibit extremely high levels of ecological adaptability through their symbiotic colonization of plants and saprophytic existence in all soil types [3]. For a long time, the functional study of *Trichoderma* spp. has been a hot topic tirelessly investigated by scientists in this field. *Trichoderma* spp. have the capacity to promote plant growth and defend various plant diseases [4], and they have established mutualistic relations with multiple plants including soybean, cucumber, tomato, etc. by colonizing the plant roots and promoting plant growth [5–7]. For instance, *Trichoderma* spp. were able to stimulate the early stages of growth in bean plants through metabolic actions, such as phosphate solubilization and siderophore and auxin production [8, 9].

Few can deny that the root system is a critical medium for the interaction between *Trichoderma* spp. and plants. The improvement in root development is frequently associated with increases in yield and biomass [10, 11]. Previously, inoculation of *Trichoderma* spp. have been reported to affect the maize root system architecture, enhancing root biomass production and increasing root hair development, which may be *Trichoderma* spp. colonize the entire root system and persist for the whole lifespan of this crop. TasHyd1, a class II hydrophobin, might mediate the attachment of spores to help *Trichoderma* spp. participate in the root colonization [12]. Moreover, Morán-Diez et al. [13] found that the *endoPG*-encoding gene was necessary for activating root colonization and inducing plant defense by *T. harzianum* T34. Although extensive studies about the role of *Trichoderma* spp. in promoting plant growth have been conducted [14], the exact molecular mechanisms that govern the recognition and association between *Trichoderma* and plant roots are still far from conclusive.

A unique gene, *swollenin*, first discovered in *Trichoderma reesei*, found synergistically hydrolyze cellulosic substrates [15, 16], little progress has a clear molecular mechanism. *T. guizhouense* NJAU4742 isolated from mature compost and stored in our laboratory was studied, and the genomic annotation results (GCA_002022785.1) indicate the presence of the *Tg*SWO gene. The sequence analysis of *Tg*SWO showed that it was similar to the plant expansins (EXPB/EXPA) [17]. Massive studies found that plant expansion proteins played a critical role in multiple aspects of plant growth and development [18], such as root formation [19–23] and responses to abiotic stresses [24].

In this study, new insights into the molecular mechanism by which NJAU4742 strain promoted cucumber growth and the functional characterization of the *Tg*SWO were deeply investigated, and the function of the *Tg*SWO gene in mediating cucumber growth was also evaluated using various mutants. Additionally, we found that *Tg*SWO could promote the cucumber growth, mainly depending on the YoaJ domain, by modifying the root architecture and increasing the root colonization. Overall, the results of this study indicate for the first time that *Tg*SWO could target the root cell wall and thus are expected to benefit the development of PGPM-plant interactions.

## Results

### *Tg*SWO was induced by the cellulose component of the root

**Fig. 1:**
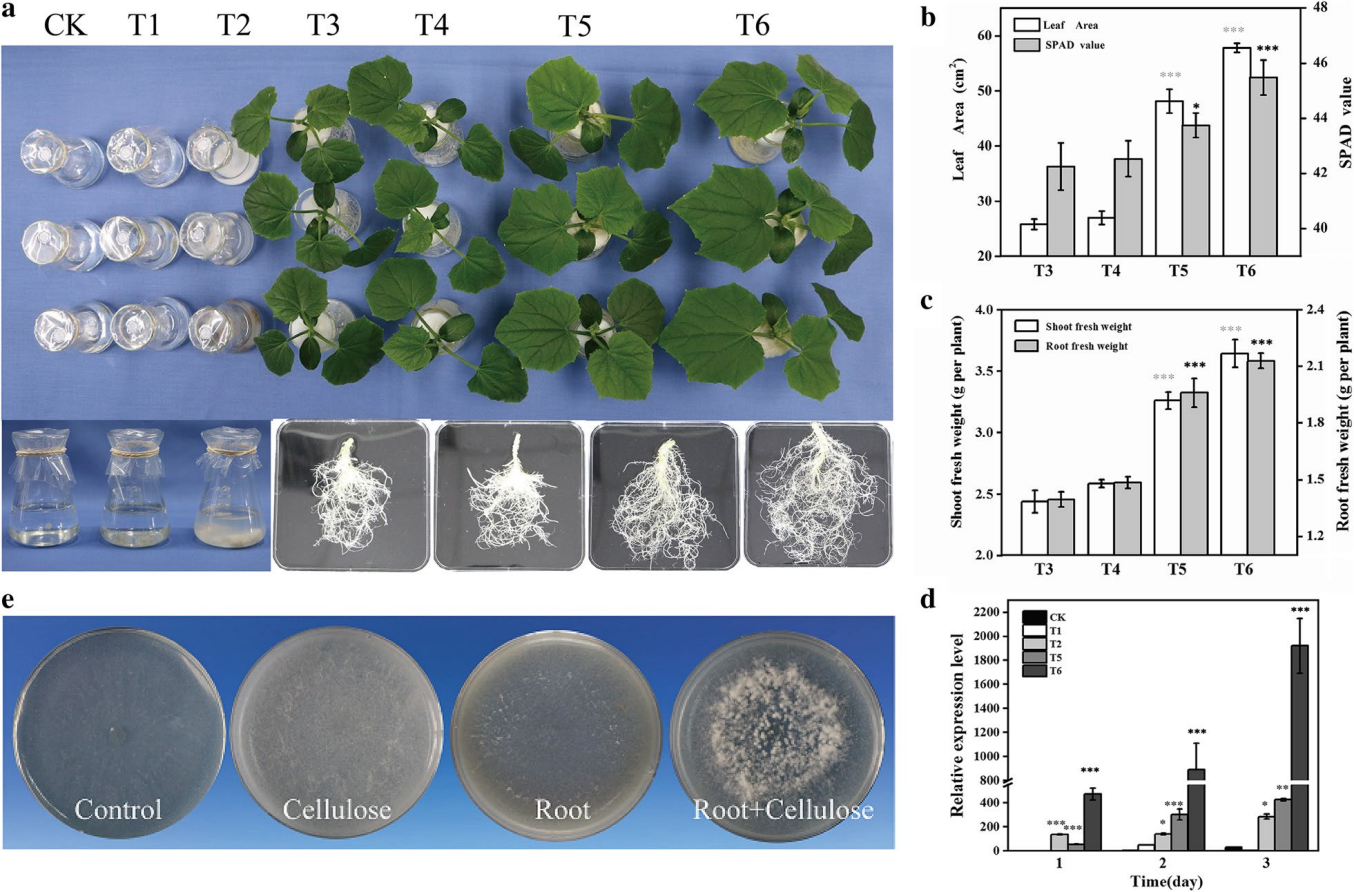
The effects of *Tg*SWO from *Trichoderma guizhouense* NJAU4742 on the growth of cucumber seedlings. **a** The growth of cucumber seedlings under different conditions, and the coculture time with NJAU4742 strain was 5 days. CK (the PGM medium + NJAU4742); T1 (the PGM medium + 1% glass fiber + NJAU4742); T2 (the PGM medium + 1% cellulose + NJAU4742); T3 (PGM medium + cucumber seedlings); T4 (PGM medium + cucumber seedlings + 1% cellulose); T5 (PGM medium + cucumber seedlings + NJAU4742); T6 (PGM medium + cucumber seedlings + 1% cellulose + NJAU4742). **b** The Leaf Area and SPAD values of cucumber seedlings in different treatments. **c** The Root fresh weight and Shoot fresh weight values of cucumber seedlings in the different treatments. **d** The expression of *Tg*SWO from NJAU4742 strain under different treatments. All data were normalized to the housekeeping gene Te*f1*, which was used as an internal control and amplified under the same conditions, and the CK treatment (1 day) with the lowest expression set to 1. Each bar represents the mean of three biological replicates with SE, and statistically significant difference as evaluated by one-way ANOVA: *P < 0.05; **P < 0.01, ***P < 0.001. **e** Growth conditions of NJAU4742 strain in different mediums with root and cellulose as sole carbon sources

To study whether *Tg*SWO is involved in the interaction between *Trichoderma* and cucumber, NJAU4742 strain was inoculated into different treatments and grew for 5 days. During the 5 days of NJAU4742 inoculation, the growth of cucumber was the best in T6, followed by T5, and no significant differences were observed between T3 and T4 (Fig. 1a). As shown in Fig. 1b and c, the Leaf Area, SPAD, root fresh weight and shoot fresh weight of cucumber in T4 had increased by 86.6%, 3.6%, 40.3%, and 33.6% on the 5th day, respectively, in comparison with T3. Interestingly, compared to T3, the increased percentages of these parameters mentioned above of cucumber were 123.9%, 7.7%, 52.5%, and 49.4% on the 5th day, respectively, for T6, whereas no significant differences were observed between T3 and T4.

The *Tg*SWO gene expression levels of NJAU4742 strain in the different treatments were evaluated using quantitative reverse transcription PCR (qRT-PCR). The results showed that the expression of *Tg*SWO gene was most abundant in T6, followed by T5 and T2, except for CK and T1, in which *Tg*SWO gene remained at a low level during the cultivation process (Fig. 1d). Notably, the expression of *Tg*SWO gene on the 3rd day in T6 was approximately 63-fold and fourfold higher than that in CK and T5, respectively (Fig. 1d). In addition, the cultivation results of cellulose utilization in the plate indicated that NJAU4742 strain exhibited an excellent growth condition on root + cellulose medium and root medium in comparison with the cellulose medium (Fig. 1e). The results obtained here suggest that the *Tg*SWO gene could be induced by the cellulose component of the root and that it might also participate in the promotion of growth in cucumber.

### Structural analysis of *Tg*SWO and expression of FRDP of *Tg*SWO

**Fig. 2:**
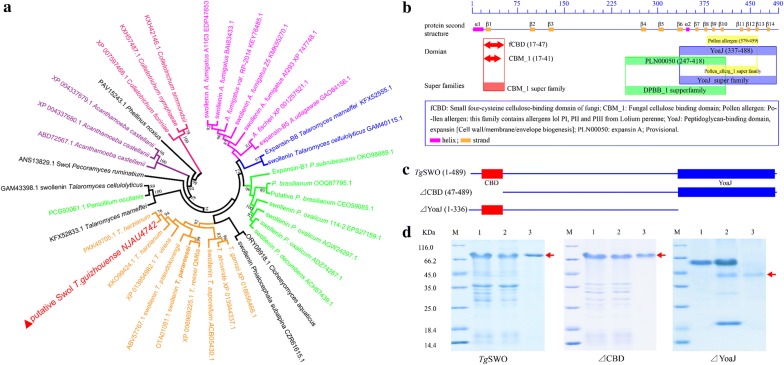
Architecture, phylogenetic analysis of *Tg*SWO proteins, and the expression of the different FRDP. **a** Phylogenetic analysis results of the *Tg*SWO protein from NJAU4742 compared with that from other fungi; the bootstrap values are shown at each node. **b** The analysis results of individual domains of *Tg*SWO from NJAU4742 strain, and different FRDP of *Tg*SWO are marked with different colors. **c** The diagram of the different FRDP based on *Tg*SWO; the domains are represented by colored rectangles. **d** The SDS-PAGE analysis results of recombinant *Tg*SWO, ΔCBD protein, and ΔYoaJ proteins. M was the markers; lane 1 represents the cell lysate; lane 2 indicates the effluents; and lane 3 refers to the electrophoretic proteins

The phylogenetic analysis between *Tg*SWO from NJAU4742 strain and Swollenin proteins secreted by other fungi showed marked phylogenetic divergence between *Tg*SWO of NJAU4742 strain and Swollenin proteins synthesized by other *Trichoderma* spp. as well as other fungi (Fig. 2a). The results obtained in this study showed that the *Tg*SWO of NJAU4742 strain was more closely related to the *T. harzianum*. Moreover, the identification result of the heterologously expressed protein in *E. coli* through the mass spectrometry analysis showed that the purified protein was *Tg*SWO, which shared 54% similarity with the *Tg*SWO from *T. pseudokoningii* (ABV57767.1) (Additional file 1: Fig. S2; Table S2).

The domains contained in *Tg*SWO were analyzed through the NCBI website (https://www.ncbi.nlm.nih.gov), and the results show that the *Tg*SWO included a carbohydrate-binding domain (CBD, residues 17–47), a PLN00050 domain (DPBB, residues 247–418) and a YoaJ domain (residues 337–488) (Fig. 2b). Experts in the lignocellulose biodegradation fields have been familiar with the CBD domain. Nevertheless, it is a little-known fact that the YoaJ domain could also own the capacity of binding the cellulose and pectins of the cell wall. The secondary structure analysis results of *Tg*SWO showed that the YoaJ domain contained a β-sandwich fold with an Ig-like fold, resembling the CBM63 (as the founding member of a new CBM family) [25, 26] (Fig. 2b). Interestingly, the YoaJ domain shared a high homology with EXLX1 from *Bacillus subtilis,* a bacterial expansin promoting root colonization of maize [27]. Based on the results described above, the *Tg*SWO shared two cellulose-binding functional regions present in the CBD and YoaJ domains, whereas the YoaJ domain was able to further act on the pectins contained in the root cell wall.

**Fig. 3:**
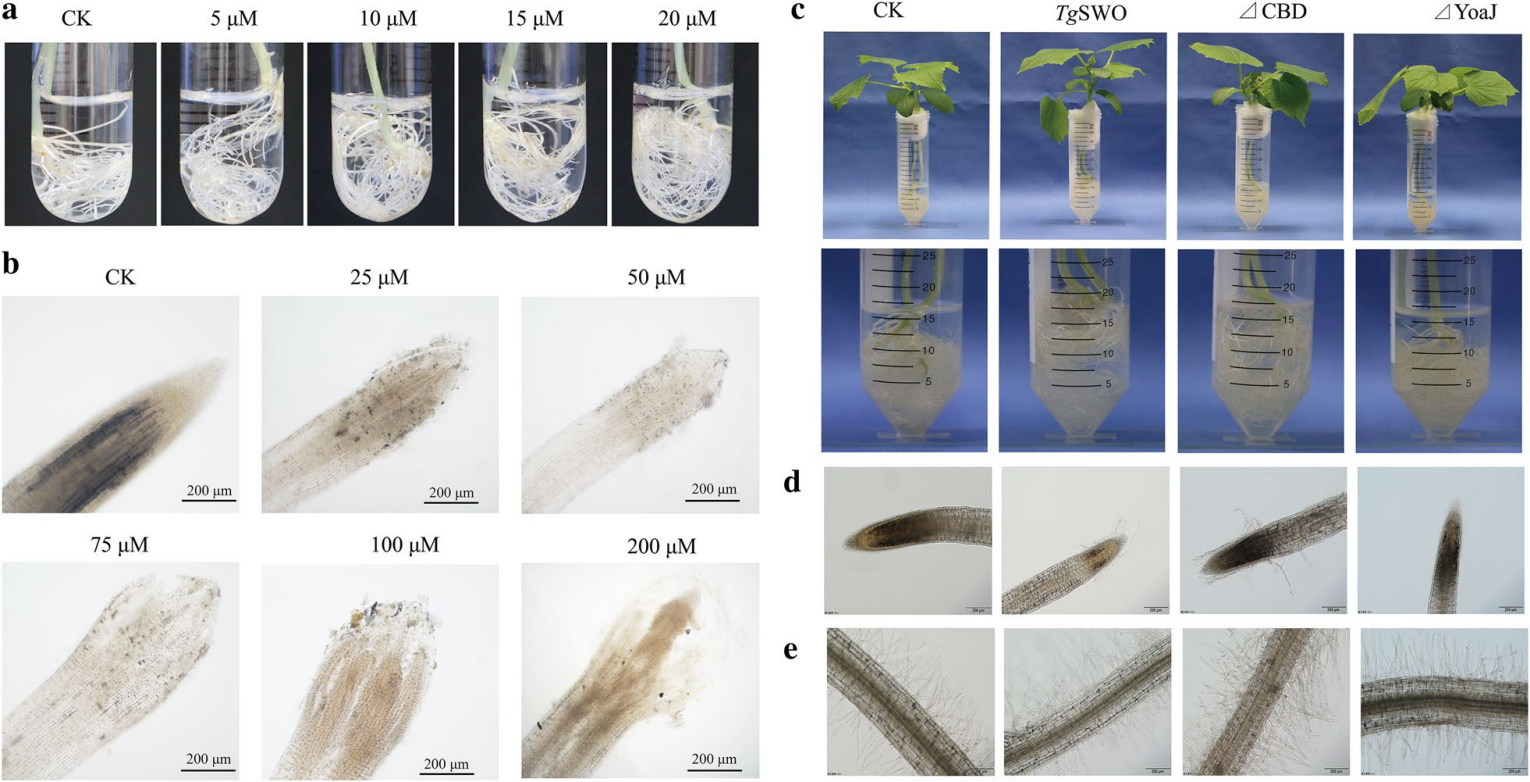
The effects of the different FRDP on the root architecture of the cucumber seedlings. **a** Effect of *Tg*SWO on the root architecture of the cucumber. Cucumber seedlings (15-day-old) grew for 48 h with 5, 10, 15, and 20 µM *Tg*SWO, respectively, and CK without *Tg*SWO was also performed under the same conditions as mentioned above. **b** Effect of *Tg*SWO on the root morphology of the cucumber seedlings. Cucumber seedlings (15-day-old) grew for 48 h with 25, 50, 75, 100 and 200 μM *Tg*SWO, respectively, and CK without *Tg*SWO was as indicated above. **c** Effect of *Tg*SWO and the different FRDP on the growth of cucumber seedlings. Cucumber seedlings grew for 48 h with SWO (treated with 15 µM *Tg*SWO), ΔCBD (7 µM ΔCBD protein) and ΔYoaJ (10 µM ΔYoaJ protein), respectively, and CK without any proteins was also performed under the same conditions as mentioned above. **d**, **e** Effect of *Tg*SWO and different FRDP on the root morphology of the root tips and root hairs of cucumber seedlings after being treated with different FRDP. Each treatment had three biological replicates, and each replicate was represented by at least 15 independent roots

To identify the critical functional region of *Tg*SWO, a series of FRDP were heterologously expressed in *E. coil* BL21 to reveal the actual functionality of *Tg*SWO. Figure 3c provides a diagram of the FRDP, and the ΔCBD was the protein lacking in the CBD domain (residues 17–47), whereas ΔYoaJ was the protein lacking in the YoaJ domain (residues 337–488). The SDS-PAGE analysis results indicated that *Tg*SWO (488 amino acid residues, 67.15 kDa), ΔCBD (452 amino acid residues, 64.38 kDa) and ΔYoaJ (336 amino acid residues, 50.65 kDa) were successfully expressed; and all proteins were electrophoretically pure (Fig. 2d–f); and approximately 1 mg of purified recombinant protein was obtained per liter of bacterial culture.

### Modification of root architecture by *Tg*SWO from NJAU4742 strain

To more closely evaluate the effects of *Tg*SWO on cucumber growth, the cucumber growth promotion by *Tg*SWO was further examined through the pretreatment of cucumber roots with different concentrations of the *Tg*SWO. Interestingly, the *Tg*SWO could significantly increase the cucumber root number (Fig. 3a). Compared with the CK, the root tips of 15 μM and 20 μM was significantly increased by 38.0% and 76.2%. Furthermore, the root number, determined by comparison to CK, was positively related to the protein concentrations, ranging from 5 to 20 μM. In particular, the 20 μM could increase the root surface area and root length by 66.1% and 46.2%, respectively, compared with the CK (Additional file 1: Fig. S5). On the other hand, the root morphological parameters of the root tips and root hairs were observed by optical microexamination, with specific changes including root tips swelling, cell wall layers sloughing off, and secretions of mucigel being noted (Fig. 3b). The results indicate that root architecture and morphology were modified by *Tg*SWO (Fig. 3a, b). Additionally, CLSM observations showed that an enormous number of NJAU4742 strain spores were attached to the cucumber root surfaces pretreated with *Tg*SWO (15 μM) (Additional file 1: Fig. S3), which might be anticipated to benefit the root colonization of PGPM. From the evidence mentioned above, we deduced that the *Tg*SWO could change the root architecture, especially root hair number, which might promote the growth of cucumber.

Subsequently, the critical functional region of *Tg*SWO was further investigated by treating the cucumber seedlings with different FRDP. As shown in Fig. 3c and e, the *Tg*SWO, and ΔCBD proteins significantly promoted the growth of cucumber seedlings as well as increasing the root number. Curiously, the ΔCBD treatment could substantially modify the cucumber root architecture, as reflected by cucumber roots (root tips and root hairs), in comparison with the CK and ΔYoaJ treatments (Fig. 3d, e). Compared with the CK, the total root length of ΔCBD treatment was significantly increased by 28.0% (Additional file 1: Fig. S6). Moreover, the ΔCBD protein exhibited an even more apparent effect on root tip swelling and cell wall layer sloughing off than that of the *Tg*SWO. However, no significant differences in the root architecture were detected after treatment with ΔYoaJ protein in comparison with CK. In summary, these data suggested that the YoaJ domain, rather than the CBD domain, might be the critical functional structure unit of *Tg*SWO.

### *Tg*SWO acts on the root cell wall via its YoaJ domain

**Fig. 4:**
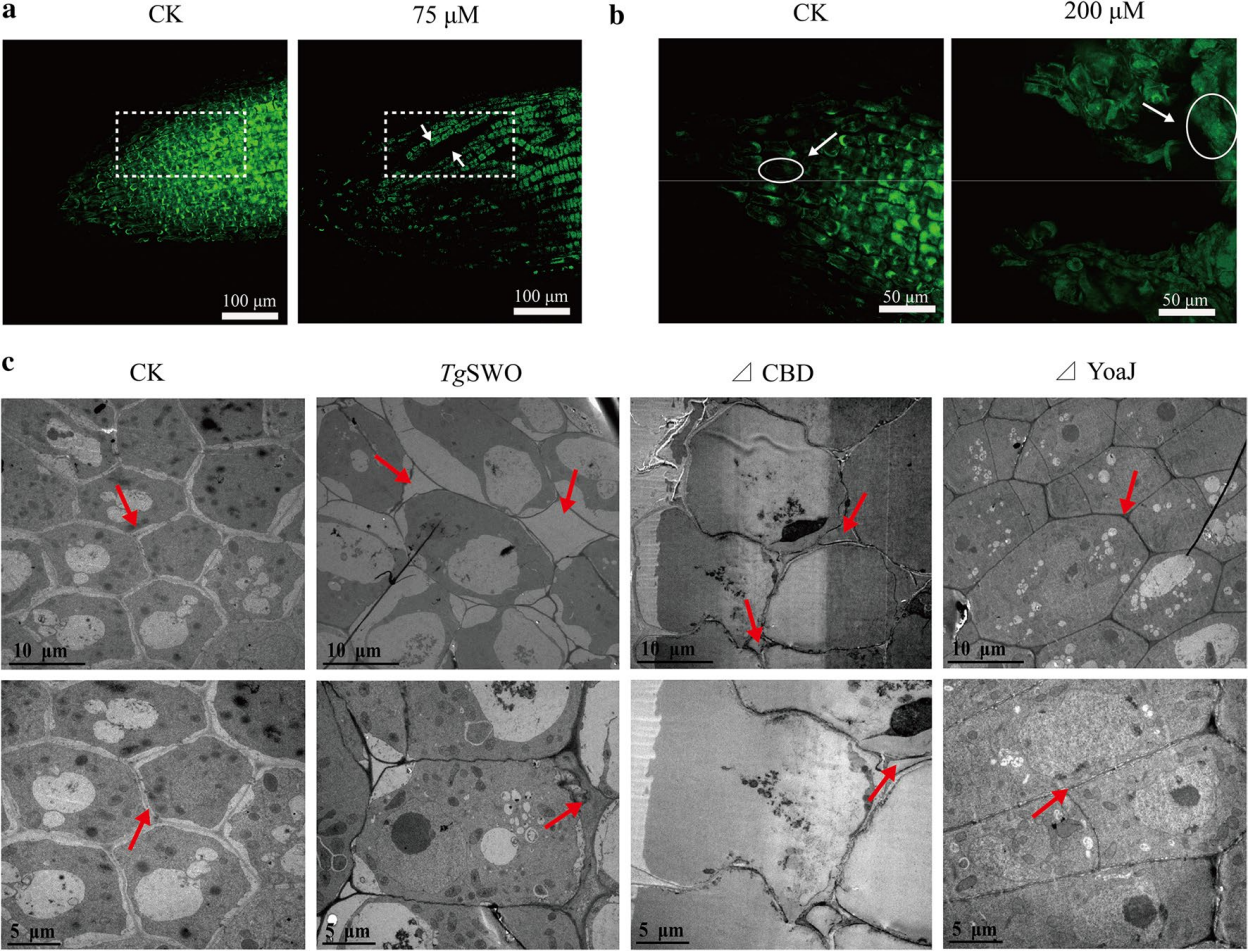
The effects of *Tg*SWO and the different FRDP on cell architecture in the various treatments. **a** The CLSM analysis results of the root tips from the CK and 75 μM *Tg*SWO treatments, and the white dotted squares represent the change of cell arrangement. **b** The CLSM analysis results of the root tips from the CK and 200 μM *Tg*SWO treatments, and the white dotted squares represent the change in cell size. Graphics were representative of at least 15 independent roots for every treatment. **c** The slice analysis results of the root tips in different treatments after treatment with *Tg*SWO and other FRDP for 48 h. SWO (15 µM), ΔCBD (7 µM), and ΔYoaJ (10 µM) were used to treat the cucumber seedlings; CK was without any protein; and the red arrow indicates the cell wall intracellular space

*Tg*SWO significantly influenced the cucumber apical organization of tightness, as indicated by the CLSM results, which showed the cucumber root tip cells loosely arranged in the 75 μM treatment (Fig. 4a). However, the root tip cells in the 200 μM treatment were expanded and large, even arranged irregularly (Fig. 4b), which might indicate that an optimal concentration exists during the action on cucumber roots by *Tg*SWO. Furthermore, the effect of the different FRDP on root cell architecture was evaluated by slice analysis (Fig. 4c). Compared with CK, the cell and intercellular space were significantly more extensive in the *Tg*SWO and ΔCBD treatment. However, the ΔYoaJ treatment did not affect the cell arrangement or cell compared with CK. These results illustrated that *Tg*SWO, as well as ΔCBD proteins, could expand the cells, loosen the root cell walls and increase the wall intracellular space which might benefit the cucumber growth and the root colonization of NJAU4742 strain. Additionally, the results mentioned above suggest that the YoaJ domain was the critical functional and structural unit of *Tg*SWO. *Tg*SWO might modify the root cell architecture through the function of the YoaJ domain and subsequently might promote the growth of cucumber.

### Involvement of *Tg*SWO in the process of root colonization of NJAU4742 strain

**Fig. 5:**
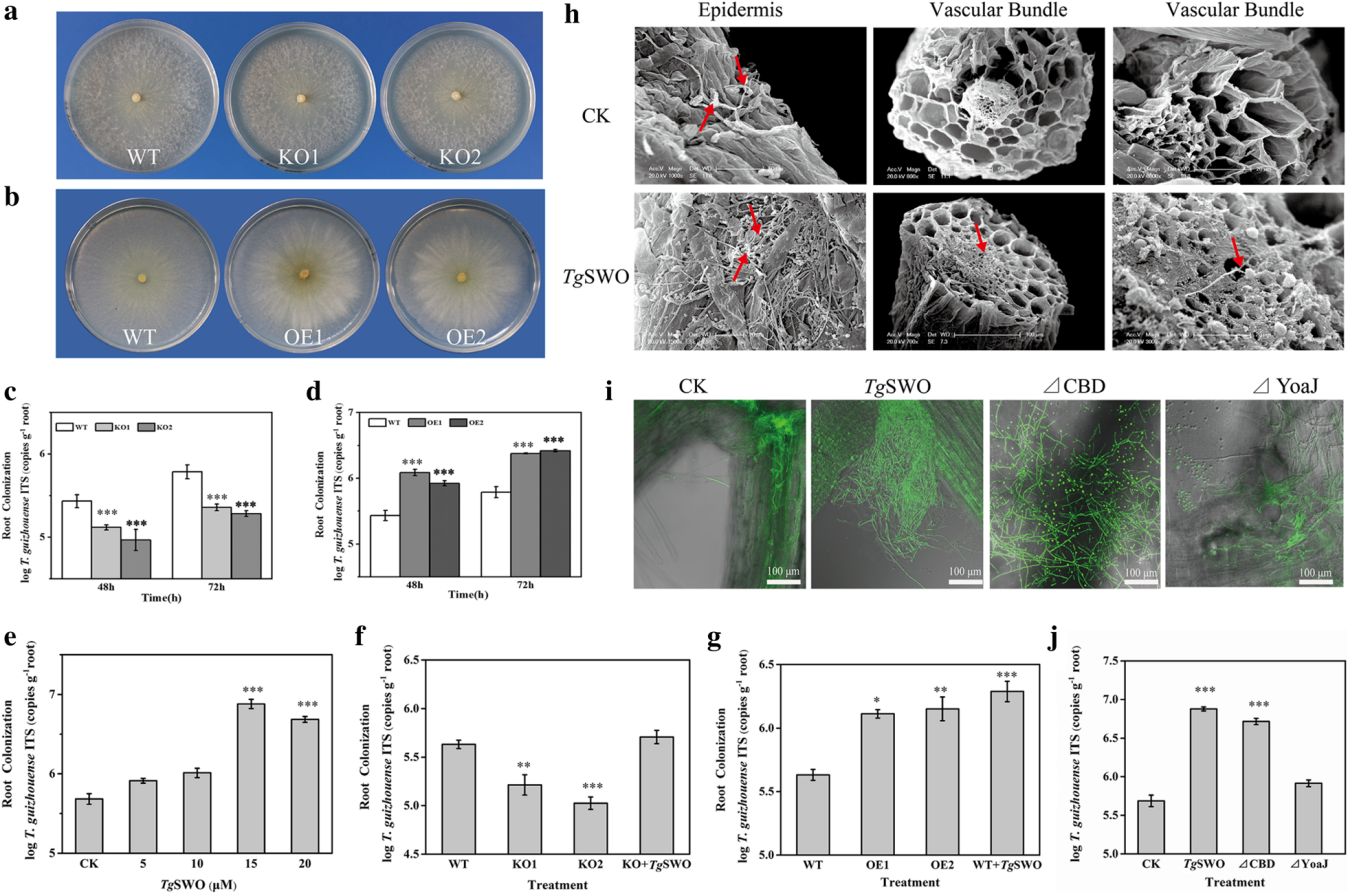
The colonization of NJAU4742 strain on cucumber roots after being treated by various FRDP. **a** The morphological characteristics of *Tg*SWO knockout mutants; KO1 and KO2 were two relatively independent mutants. **b** The morphological characteristics of *Tg*SWO overexpressed mutants; OE1 and OE2 were two relatively independent mutants. **c** The colonization of the KO mutants (KO1 and KO2) on the roots of the cucumber seedlings, with the wild-type used as a control, and each bar represent the mean of three biological replicates with SE. **d** The colonization of the OE mutants (OE1 and OE2) on the roots of the cucumber seedlings, with wild-type used as the control, and each bar representing the mean of three biological replicates with SE. **e** The colonization of NJAU4742 strain on the cucumber roots after being treated by *Tg*SWO at different concentrations (5, 10, 15, and 20 μM), and CK was without *Tg*SWO. **f** Comparison of the colonization of different strains on the cucumber roots; KO + *Tg*SWO indicates *Tg*SWO was added exogenously to the KO1 mutant 48 h after being inoculated. **g** Comparison of the colonization of the different strains on the cucumber roots, and WT + *Tg*SWO indicates *Tg*SWO was added exogenously to the wild-type 48 h after inoculation. **h** SEM observations of root colonization of NJAU4742 strain. **i** CLSM analysis results of GFP-marked NJAU4742 strain colonizing the cucumber roots in *Tg*SWO (15 µM), ΔCBD (7 µM), ΔYoaJ (10 µM), and CK was without any proteins. **j** The quantification of root colonization of the wild-type under the different treatments. Each bar represents the mean of three biological replicates with SE. Statistically significant difference as evaluated by one-way ANOVA: *P < 0.05; **P < 0.01, ***P < 0.001

The effects of KO and OE of *Tg*SWO mutants on root colonization were evaluated by the inoculation of different strains into the 15-day-old cucumber seedlings to illuminate the possible roles *Tg*SWO plays during the root colonization process of NJAU4742 strain. The growth characterization of the WT, KO, and OE mutants was compared to clarify the function of *Tg*SWO, and the results indicate that no significant difference occurred in the growth among these strains (Fig. 5a, b). qRT-PCR analysis results showed that the expression of *Tg*SWO gene in OE increased such that the OE1 and OE2 increased by 23.8 and 16.7-fold, respectively, compared to the WT (Additional file 1: Fig. S1). Simultaneously, the expression of *Tg*SWO could significantly affect the colonization of NJAU4742 strain on cucumber root. As shown in Fig. 5c, significant differences were detected between the WT and KO regarding root colonization at 48 and 72 h after inoculation. Compared with the WT, the colonization number of KO1 and KO2 was significantly reduced by 52.1% and 65.2% at 48 h and by 63.1% and 69.1% at 72 h, respectively. Additionally, the OE1 and OE2 could colonize more on the cucumber roots as indicated by 4.5 and 3.1-fold at 48 h and by 3.8 and 4.2-fold at 72 h, respectively, showing an increase in comparison with the WT (Fig. 5d). The results noted above suggest that *Tg*SWO could increase the colonization of NJAU4742 strain on cucumber root without affecting the growth of NJAU4742 strain.

Because the expression level of *Tg*SWO was closely related to the root colonization of NJAU4742 strain, the function of *Tg*SWO was analyzed accurately (Fig. 5e). Significant differences existed in the root colonization number of NJAU4742 strain between the *Tg*SWO (with the concentrations of 15 μM and 20 μM) and CK. The root colonization number of NJAU4742 strain in 15 μM and 20 μM was approximately 16 and tenfold higher, respectively, than that of CK. We also quantified the root colonization of the wild-type strain, KO, and OE mutants through exogenous supplementation of *Tg*SWO (Fig. 5f, g), and the results indicated that the KO1 mutant in the KO +* Tg*SWO treatment (KO1 mutant inoculated in the cucumber being treated with *Tg*SWO) showed a threefold increase in the colonization number in comparison with the KO1 treatment (without *Tg*SWO). Meanwhile, the wild-type in the WT +* Tg*SWO treatment (wild-type strain inoculated in the cucumber being treated with *Tg*SWO) showed up to a fivefold increase in the colonization number when compared with the WT treatment. In addition, *Tg*SWO knockout mutants reduced root colonization, as indicated by a 61.2% and 75.1% decrease in the KO1 and KO2, respectively. However, *Tg*SWO overexpression mutants promoted root colonization, as indicated by threefold increases in both OE1 and OE2. All data obtained above clarified that *Tg*SWO could increase the root colonization of NJAU4742 strain. Interestingly, NJAU4742 not only could colonize the epidermis and the outer root cortex of the cucumber roots [28] but could also colonize the root vascular bundle after it had been treated with the *Tg*SWO (Fig. 5h), which might be anticipated to benefit the development of the microbe-plant interactions.

Subsequently, the effect of the YoaJ domain on the colonization of NJAU4742 strain was determined by observation of the rhizosphere behavior of the GFP-labeled NJAU4742 strain (Fig. 5i). The results show that massive GFP-labeled NJAU4742 strain were attached to cucumber root surfaces in the *Tg*SWO and ΔCBD treatment in comparison with the ΔYoaJ and CK treatment. The colonization number of NJAU4742 strain in the *Tg*SWO and ΔCBD treatment was 14.4 and 9.6-fold higher than that of CK, respectively, but no significant differences in the ΔYoaJ and CK treatment were detected (Fig. 5j).

### Effects of FRDP on the growth of the cucumber seedlings

**Fig. 6:**
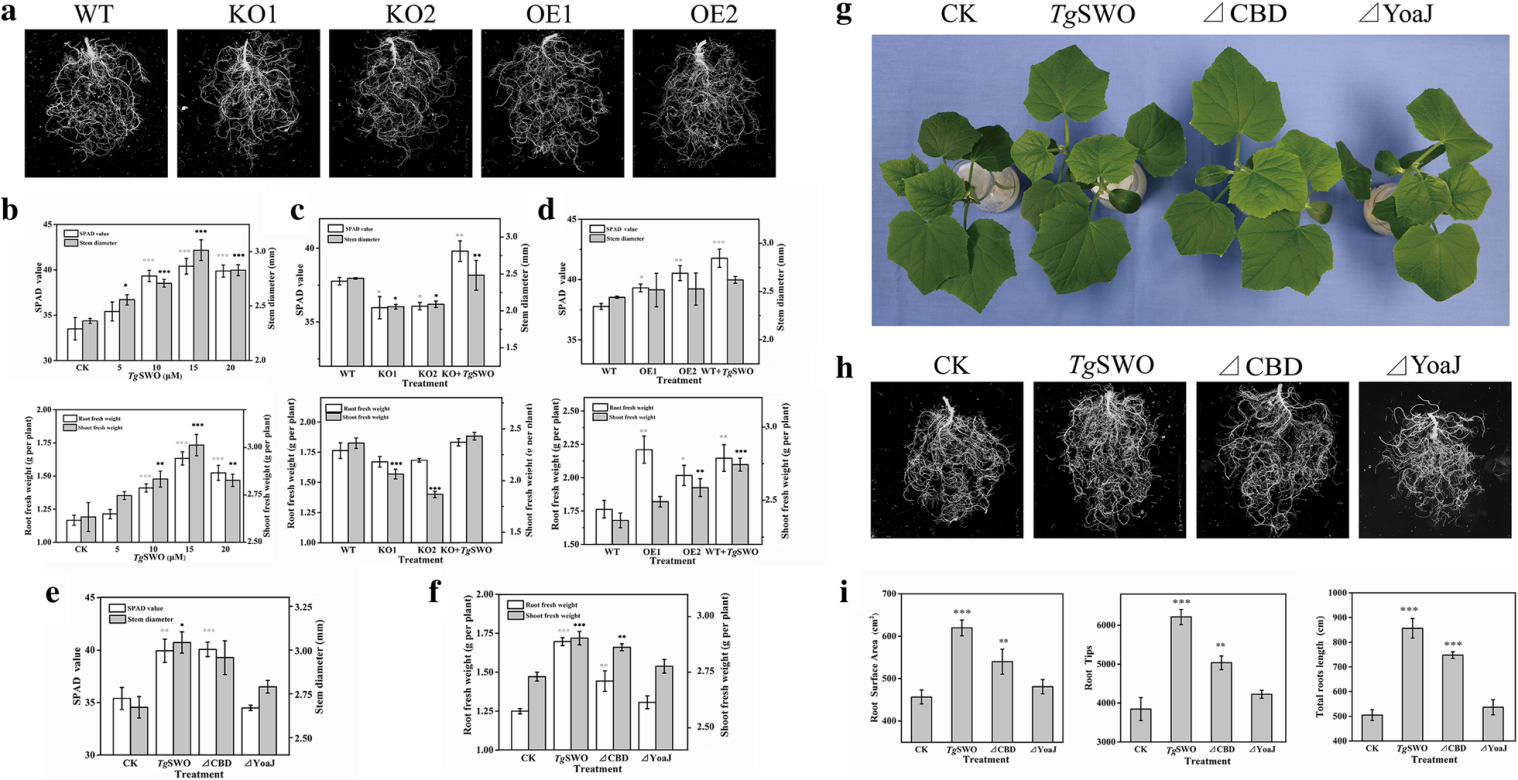
The effects of various FRDP on cucumber growth. **a** The values of the root physiological parameters of cucumber seedlings after being inoculated with KO (KO1, KO2) and OE (OE1, OE2), with wild-type used as a control. **b** The SPAD, stem diameter, root fresh weight and shoot fresh weight of cucumber seedlings after being treated by *Tg*SWO at different concentrations. **c**, **d** Comparison of the SPAD, stem diameter, root fresh weight and shoot fresh weight values of cucumber seedlings in the different treatments. **e**, **f** The effects of the various FRDP on the cucumber growth, and the treatments, which included *Tg*SWO (15 µM), ΔCBD (7 µM), ΔYoaJ (10 µM) and CK (without any proteins). **g** The growth conditions of the cucumber under the different treatments. **h**, **i **The effects of the various FRDP on the cucumber growth, and the parameters of root surface area, root tips, and total root length were determined to evaluate the growth promotion of the cucumber seedlings. All results were representative of three independent experiments, and each bar represents the mean of three biological replicates with SE, and statistically significant difference as evaluated by one-way ANOVA: *P < 0.05; **P < 0.01, ***P < 0.001

The effects of KO and OE of *Tg*SWO mutants on the cucumber growth were evaluated by inoculation of different strains into the 15-day-old cucumber seedlings. As shown in Fig. 6a, compared with the WT, the root tips and root length increased significantly by 25.2% and 19.4%, respectively, for OE1 and by 29.7% and 14.8%, respectively, for OE2. By contrast, the root indexes mentioned above decreased significantly by 3.4% and 3.7%, respectively, for KO1 and by 14.6% and 2%, respectively, for KO2, respectively (Additional file 1: Fig. S4). The results suggest that *Tg*SWO promoted the growth of cucumber root. The effects of *Tg*SWO on cucumber growth were also evaluated by determining the various physiochemical parameters of cucumber. Five days after inoculation with NJAU4742 strain, the SPAD, stem diameter, root fresh weight and shoot fresh weight increased by 20.6%, 27.5%, 39.9%, and 14.4% in the 15 μM treatment in comparison with the CK. Similarly, the increased percentages of these parameters were 19.0%, 19.8%, 30.6%, and 7.35%, respectively, in the 20 μM treatment (Fig. 6b). Furthermore, the SPAD, stem diameter, root fresh weight and shoot fresh weight of cucumber increased by 10.7%, 8.9%, 30.1%, and 18.8% in the KO +* Tg*SWO treatment, in comparison with the KO1 treatment (Fig. 6c). Similarly, compared to the WT treatment, the increased percentages of SPAD, stem diameter, root fresh weight and shoot fresh weight of cucumber were 10.6%, 21.8%, 16.1%, and 7.4%, respectively, in the WT +* Tg*SWO treatment (Fig. 6d). Meanwhile, the value of the SPAD, stem diameter, root fresh weight and shoot fresh weight in the KO1 and KO2 treatments, respectively, decreased by 4.8% and 4.5%, 4.5% and 5.5%, 21.1% and 12.7%, and 14.3% and 15.7% in comparison with the WT (Fig. 6c). However, the OE1 and OE2 showed 7.3% and 4.1%, 25.4% and 14.4%, 5.4% and 9.5%, and 3.1% and 3.6% increases in the SPAD, stem diameter, root fresh weight and shoot fresh weight, respectively, in comparison with the WT (Fig. 6d). These findings further demonstrate that the *Tg*SWO could boost the cucumber growth.

Furthermore, we also focused on whether ΔYoaJ could promote cucumber growth. As shown in Fig. 6g, the cucumber in *Tg*SWO and ΔCBD grew better than in CK or ΔYoaJ. Meanwhile, the root surface area, root tips, and root length increased by 35.7%, 61.4%, and 69.5%, respectively, in the *Tg*SWO treatment compared with the CK treatment. In particular, the ΔCBD could increase the root surface area, root tips and root length by 18.3%, 30.9%, and 48.2%, respectively, compared with the CK. Nevertheless, no significant differences in the ΔYoaJ and CK treatment were detected (Fig. 6h, i). The physiology parameters were also determined to evaluate the function of the FRDP on the cucumber growth promotion. The SPAD, stem diameter, root fresh weight and shoot fresh weight in the *Tg*SWO treatment increased by 12.8%, 15.1%, 6.3%, and 35.7% compared with the CK. Similarly, the increased percentages of the SPAD, stem diameter, root fresh weight and shoot fresh weight of the cucumbers were 13.2%, 9.6%, 4.8%, and 15.5%, respectively, in the ΔCBD treatment in comparison with CK, but no significant differences were found between the ΔYoaJ and CK treatment (Fig. 6e, f). In summary, the *Tg*SWO promoted cucumber growth mainly by YoaJ domain. Additionally, the YoaJ domain response included expanding the cell wall to modify the root architecture and promote root colonization of NJAU4742 strain.

## Discussion

The root system plays a fundamental role in plant growth [29]. Root size and architecture are the factors that determine yield performance, particularly under conditions of a variety of abiotic stresses, such as nutrient deficiency and drought [30, 31]. In this study, a newly identified expansin-like protein from NJAU4742 strain, designated as *Tg*SWO, was confirmed to be involved in the growth and development of the cucumber root system through investigations on the functions of *Tg*SWO and FRDP (ΔCBD and ΔYoaJ). Additionally, we observed modifications in the root cell wall and determined the critical functional region of *Tg*SWO. Furthermore, we have confirmed the role of *Tg*SWO in the cucumber growth promotion and root colonization of NJAU4742 strain by knockout and overexpression methods.

As a root symbiont, *Trichoderma* spp. can invade and colonize roots, thereby promoting plant growth and productivity [32]. The increased expression of the *Tg*SWO gene during symbiosis establishment in the interaction between NJAU4742 strain and cucumber confirmed this point. We also found that the *Tg*SWO of NJAU4742 strain was highly expressed under induction by cellulose [16, 33, 34]. Remarkably, *Tg*SWO of NJAU4742 strain showed the highest expression in the coculture of cellulose and cucumber roots, with cucumber showing the best growth. Furthermore, the plate culture experiment indicated that NJAU4742 strain could grow faster on the Root + Cellulose medium and the Root medium, whereas the OE mutants grew faster and exhibited denser mycelium than the KO mutants in the root medium (data not shown). Therefore, we speculated that *Tg*SWO might directly act on the cucumber roots.

Delightedly, the numbers of cucumber roots increased significantly when *Tg*SWO was expressed and used to pretreat the cucumber seedlings. Furthermore, the optical microexamination analysis showed that *Tg*SWO could change root architecture and expand root cells, especially in the root cap zones. The root cap could mediate root architectural changes [35, 36], and the mucigel consisted of highly hydrated polysaccharides that were readily degradable targets for the hemicellulases of *Trichoderma* spp. [37], which might have been responsible for the adsorption of massive spores on the root surface (Additional file 1: Fig. S3). Hence, this result indicated that *Tg*SWO might be involved in the cucumber root development and the cucumber root architectural responses to the colonization of NJAU4742 strain. Remarkably, this mechanism was different from results from a previous study in which *Trichoderma* spp. were able to promote root growth through an auxin-dependent mechanism [38]. Meanwhile, importantly, exorbitant *Tg*SWO would cause cucumber seedlings to wilt, which indicates that *Tg*SWO might have unknown functions in addition to being involved in regulating cucumber root growth.

According to our observations, *Tg*SWO might have great potential in improving root colonization. Previous research demonstrated that overexpression of the complete *Tg*SWO open reading frame could promote root colonization efficiency of *T. asperellum* [39]. Here, we heterologously expressed *Tg*SWO, and directly demonstrated that *Tg*SWO could promote root colonization. In addition, the root colonization of NJAU4742 strain and the cell intercellular gap dramatically increased when *Tg*SWO was applied exogenously, and NJAU4742 strain might colonize the root vascular bundle after being treated with *Tg*SWO, which had never been shown in *T. guizhouense* before. Outside the *Tg*SWO of *Trichoderma* spp., a handful of other organisms exist in which expansin-like sequences have been identified. The plant-parasitic roundworm *Globodera rostochiensis* could produce a functional expansin (Gr-EXPB1) to loosen cell walls to allow invasion of the host plant [40]. Kerff et al. [27] also mentioned that EXLX1 protein might also affect the root colonization of *Bacillus subtilis* by acting on the cell wall. These reports, combined with our previous results, suggest that expansin-type regions have been adopted by different microbes to enhance their interactions with plants, whether as observed in *T. guizhouense* and nematode or *B. subtilis* noted above. Our discovery that *Tg*SWO promoted root colonization of NJAU4742 strain suggested that its biological role was to enhance the plant-fungi interaction.

Cell wall is an important structure that regulates cell shape and cell proliferation, and acts as a critical role in root growth promotion. Plant expansin might act directly on regulating the growth and development of the root system by inducing cell wall extension and stimulating cell wall stress relaxation. [41–43]. Swollenin from *T. reesei* has also been shown to disrupt the *Valonia* cell wall [44]. Here, the root cell intracellular space was increased obviously, Saloheimo et al. [44] provided direct evidence that *a Trichoderma reesei* Swollenin protein with sequence similarity to the plant expansins could expand the root cell walls of the higher plant. In addition, both CLSM and cell ultrathin sections showed that the root cells became enlarged, swollen with widened intercellular space. These results also further confirm that the increase in cell size can improve the growth of the root [45]. Therefore, we suggested that *Tg*SWO might promote the growth and development of cucumber root by inducing cell wall extension during the interaction between the NJAU4742 strain and the cucumber. In addition, some hydrolytic enzymes might promote the action of the expansin indirectly by hydrolyzing the hemicellulose or pectin that reduces the size and viscosity of cell wall matrix polymers [46, 47]. Georgelis et al. [25] reported that the D2 domain (similar to the YoaJ domain of *Tg*SWO) of EXLX1 was more capable of binding to cellulose and pectin than the D1 domain (similar to CBD domain of *Tg*SWO) and the carbohydrate-binding region in D2 was identified as CBM63 [26], which also detected in the YoaJ domain of *Tg*SWO. Specially interesting, the ΔCBD protein, though not the ΔYoaJ protein, could even exhibit a synergistic effect to loosen the root cell walls. Moreover, the ratios between the cell wall polymers (cellulose, pectin, and hemicellulose) were essential to maintain the structural integrity of the cell wall, and the precise sites of expansin could affect the distribution of cell wall polymers [48]. Our results also clearly show that the ΔCBD protein could show greater activity for inducing cell wall extension. A possible explanation was that competition for substrate might exist between the CBD domain and the YoaJ domain, probably for cellulose, and in the *Tg*SWO, the ability of pectin of the YoaJ domain to bind was limited by the traction power of the CBD domain. Hence, *Tg*SWO’s action could be determined by its wall-binding properties, which might be mainly mediated by YoaJ domain.

**Fig. 7:**
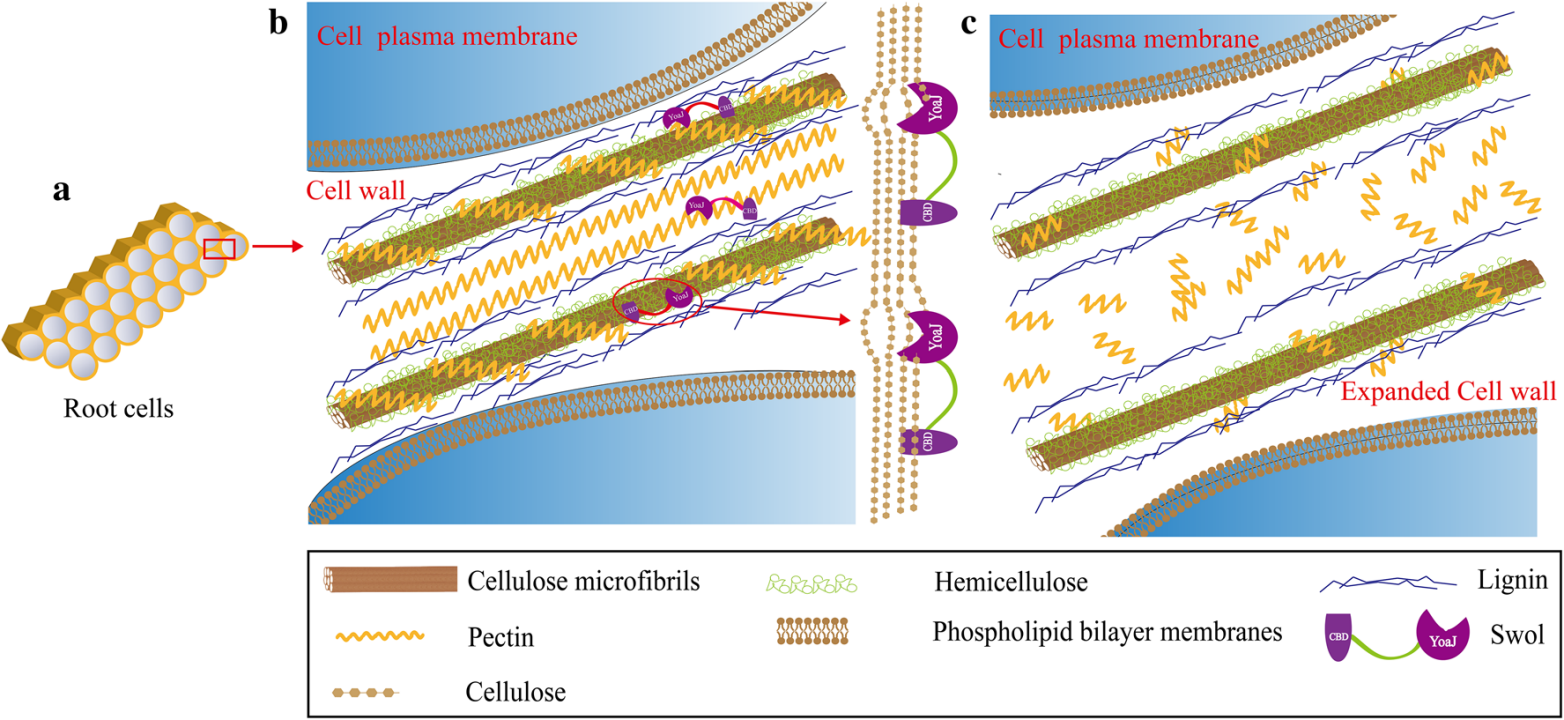
Schematic description of *Tg*SWO from NJAU4742 strain involved in expanding the cell wall. **a** A diagrammatic sketch of the root cell; **b** the schematic diagram of action taken by *Tg*SWO from NJAU4742 strain; *Tg*SWO was able to expand the cell wall in two different ways, which included acting on the cellulose and pectin, **c** the schematic diagram of cell structure after treatment of plants by *Tg*SWO

In view of previous studies and the results obtained here, we developed a schematic representation how *Tg*SWO destructed the root cell walls (Fig. 7a–c), and the exact mechanism is described below in detail. The CBD and YoaJ domain of *Tg*SWO bind to the cellulose microfibrils in the cell walls and the pectin in the cell intercellular layer, and these components are necessary for the composition of the cell walls and the arrangement of the cell [49, 50]. The YoaJ domain guided the *Tg*SWO to these particular sites, perhaps in conjunction with the cooperative action of the CBD domain, and the YoaJ domain might be mainly needed to loosen the cellulose–cellulose connections or binding to pectin, by a physicochemical process yet to be confirmed (Fig. 7b). Furthermore, we found the YoaJ domain was also unusual for *Tg*SWO of NJAU4742 strain, compared with other species including *T. parareesei* (OTA01081.1), *T. reesei* (XP_006969225.1)*, T. pseudokoningii* (EU137149.1), etc. [51–53].

Plant growth promotion often has been observed in response to *Trichoderma* spp. colonization [54, 55]. In our study, *Tg*SWO and the ΔCBD protein, but not the ΔYoaJ protein, exhibited the more powerful capacity to promote NJAU4742 strain to colonize on the cucumber roots. Moreover, the root and shoot of the cucumber increased dramatically after the exogenous application of the *Tg*SWO or ΔCBD proteins. These results suggest that successfully ensuring beneficial effects in practice required to reach the fundamental colonization level of *Trichoderma* spp. on plant roots [37, 56]. Therefore, the schematic in the graphical abstract describes how *Tg*SWO further enhances the positive interactions between *Trichoderma* spp. with plant roots, and consequently, was able to promote cucumber growth. *Tg*SWO stimulated the cucumber to produce root-derived nutrients, such as mucigel, which might attract NJAU4742 strain, to establish themselves in the rhizosphere [37], and then *Tg*SWO further acted on the root cell wall to increase root colonization by NJAU4742 strain and the growth of the cucumber root.

## Conclusions

This study demonstrated that *Tg*SWO might mainly depend on the YoaJ domain, whose response expands the cell wall, thereby modifying the root architecture, increasing root colonization of NJAU4742 strain, and promoting cucumber growth. A better understanding of the molecular mechanism of *Tg*SWO-mediated wall loosening may provide new strategies for enhancing the effectiveness of PGPM, which will increase the colonization of PGPM and offer a novel approach for constructing unique nanocellulosic structures through an understanding of the mechanism of *Tg*SWO disruptions of cellulose.

## Experimental procedures

### Fungal cultivation and cucumber planting

*Trichoderma guizhouense* NJAU 4742 (CGMCC NO.12166, China Microbial Culture Collection Committee General Microbiology Center) was stored in Jiangsu Key Laboratory for Organic Solid Waste Utilization. The strain was grown in potato dextrose agar (PDA) medium (Difco Laboratories, Detroit), and the conidial suspension of NJAU4742 strain was prepared as described by Yang et al. [57]. The germinated spore was prepared as described by Yedidia et al. [28], and a final concentration of 10^7^ germinated spores mL^−1^ was used in the inoculation experiments. The growth of cucumber seedlings (*Cucumis sativus L. cv* JinChun-No. 4 obtained from Tianjin Cucumber Research Center, China) was undertaken and plant growth medium (PGM) was prepared as previously described with some modifications. The PGM liquid medium were improved with 1/3 Hoagland nutrient solution [8, 58]. The germinated spores of NJAU4742 strain were added to the PGM liquid medium with 15-day-old seedlings to a final concentration of 10^5^ germinated spores mL^−1^ under aseptic conditions. The cucumber seedlings were grown under glasshouse conditions (50 rpm, 80% relative humidity, 28 °C and a photoperiod of 14 h light and 10 h dark) for 5 days, after which various parameters including leaf area, shoot fresh weight and relative expression level of *Tg*SWO were detected.

### RNA extraction and qRT-PCR analysis

Total RNA from the *Trichoderma* mycelium was extracted as described by Brotman et al. [39], the qRT-PCR analysis was performed according to Viterbo et al. [59], and the expression of the *Tg*SWO gene was performed using the SYBR^®^ Premix Ex Taq™ II (RR820A, Takara, Dalian, China) as described by Viterbo et al. [60]. Primers for the qRT-PCR analysis of *Tg*SWO and *Tef1* (GenBank accession no. Z23012) are listed in Table S1, and data were expressed as the means of three replicates. This method calculated the relative expression of the specific gene by referring to formula 2 (− ΔCT), where ΔCT = CT of the specific gene-CT of the reference gene.

### Vector construction and transformation

Based on the principle of homologous recombination protocol, knockout fragments of *Tg*SWO gene fragments were constructed as follows: a fragment of 1200 bp upstream from *Tg*SWO was amplified by primers swo-upF and swo-upR (Additional file 1: Table S1), and a 1200 bp fragment downstream from *Tg*SWO was obtained by using swo-dF and swo-dR as primers (Additional file 1: Table S1), whereas primers HygB-F and HygB-R (Additional file 1: Table S1) were used to amplify the hygromycin resistance fragment from pUCcDNA1 (provided by the Microbiology group, University of Vienna, Austria). The three fragments were ligated using the overlapping-PCR technique according to the instructions of CloneAmp HiFi PCR Premix (Clontech), and the corresponding sequences are displayed in Additional file 1: Table S1. The *Tg*SWO knockout fragment was purified using E.Z.N.A.^®^ Cycle Pure Kit (D6492-01, OMEGA, USA). The transformation of the knockout fragment into NJAU4742 strain protoplasts with polyethylene glycol (PEG) was carried out [61, 62]. The colonies formed on the antibiotic screening plate were selected as the *Tg*SWO-knockout transformants of *T. guizhouense* NJAU4742 strain (Additional file 1: Fig. S1). The southern experiments were performed following standard procedures [63]. The restriction fragments were covalently bound to a Nylon + membrane (Boehringer Mannheim, Indianapolis, IN, USA), and the membrane was hybridized with probe and exposed using standard procedures according to the manufacturer’s guidelines.

Primers with restriction enzyme sites of *Nco*I and *Bst*EII were designed based on the restriction enzyme sites of *Tg*SWO and the pCAMBIA-1302 vector purchased from Addgene. The *Tg*SWO fragments were amplified using PrimeSTAR GXL DNA Polymerase (R050Q, Takara, Dalian, China) with the cDNA of NJAU4742 strain as a template, and then the fragments were inserted into the pCAMBIA-1302 vector to form the recombinant pCAMBIA1302-*swol* using a T4 DNA Ligase Kit (D2011A, Takara, Dalian, China) to perform a ligation reaction. The pCAMBIA1302-*swol* was transferred into *A. tumefaciens* EHA105 (stored in our Lab) using Gene Pulser Xcell™ (Bio-Rad, USA) according to Mahmood et al. [64], and the positive transformants were selected from the antibiotic medium. *Agrobacterium tumefaciens*-mediated transformation (ATMT) was carried out according to Yang et al. [65], with some modifications. A total of 100 μL of the *Agrobacterium* culture (1 × 10^8^ CFU) was mixed with 100 μL of NJAU4742 strain conidia (1 × 10^7^ conidia) and spread onto a filter placed on an IMAS agar plate. Plates containing the filters were incubated according to Yang et al. [65], and screening validation was used to obtain the overexpression transformants of *T. guizhouense* NJAU4742 strain. The expression of the *Tg*SWO gene in wild-type strain NJAU4742 strain, knockout mutants (KO) and overexpression mutants (OE) were analyzed through RT-PCR using Q-swoF and Q-swoR designed based on the full length of *Tg*SWO cDNA (Additional file 1: Table S1). Expression of the *Tg*SWO gene was expressed as the relative expression level by comparing with the wild-type strain by using the same primers described above.

### The expression and purification of functional regional deletion proteins

The *Tg*SWO gene cDNA sequence of *T. guizhouense* NJAU4742 strain was obtained by searching the whole genome database of *T. guizhouense* NJAU4742 strain (http://bioinfo.njau.edu.cn/tgn4742/). To identify the functional regions of *TgSWO*, different functional region deletion proteins (FRDP), including complete TgSWO, ΔCBD (without cellulose binding domain, lacking residues 1–47) and ΔYoaJ (without YoaJ, lacking residues 316–467) were expressed with an *E. coli* expression system. The gene sequence, consisting of the codons for *Tg*SWO, ΔCBD, and ΔYoaJ were synthesized by GenScript (Nanjing, China). Afterward, the integrated genes were ligated into the pET32a (+) vector. The recombinant vectors pET32a-*swol*, pET32a-ΔCBD, and pET32a-ΔYoaJ were transformed into *E. coli* BL21 cells using the calcium ion method and then plated on the ampicillin-containing (50 μg mL^−1^) agar plates. Single colonies were screened for successful ligation events by restriction digestion and DNA sequencing (GenScript, Nanjing, China).

The positive transformants containing recombinant plasmids were inoculated into 100 mL LB with 100 μg mL^−1^ ampicillin and were shaken, and IPTG was added to a final concentration of 0.5 mM to induce protein expression. The supernatants and the insoluble pellet of the cell lysate were investigated for the expression forms of the recombinant fusion proteins by SDS-PAGE analysis. The proteins were purified using amylose resin (NEB). The protein concentration of the final purified proteins was analyzed using a BCA kit (Dingsi, Beijing, China), and the purified proteins were stored at − 80 °C for the subsequent experiments.

### The functional analysis of the recombinant proteins on the plant growth promotion and increasing the root colonization of NJAU4742 strain

Cucumber seedlings (15-day-old) were treated according to Wassenberg et al. [66] as described below: without *Tg*SWO protein in PGM (CK); 15 μM TgSWO diluted in PGM (SWO); 7 μM ΔCBD diluted in PGM (ΔCBD); and 10 μM ΔYoaJ diluted in PGM (ΔYoaJ). After 48 h, the seedlings were inoculated with 1 × 10^5^ spores mL^−1^ of NJAU4742 strain, and colony proliferation was assayed after 3 days as described by Brotman et al. [39]. The cucumber seedlings were harvested after 5 days, and the growth indexes were detected according to Zhang et al. [67]. Assessment of the colonization of NJAU4742 strain on the cucumber root was performed as described by Zhang et al. [67] using quantitative fluorescence PCR. The primers UTF and ITS2P (Additional file 1: Table. S1) designed by Zachow et al. [68] were used to amplify a 158-bp fragment from ITS region. The results of the quantification were analyzed with the 7500 Real-time PCR system SDS Software version 1.4 (Applied Biosystems) according to Lopez-Mondejar et al. [69] and Huang et al. [70].

### Transmission electron microscope and confocal laser scanning microscopy observation

In the axenic, hydroponic, growth systems, the root samples were monitored for 48 h after being treated with different functional region deletion proteins (FRDP), including *Tg*SWO, ΔCBD, and ΔYoaJ. The collected root tips were cut into 3–4 mm lengths and then visualized using an Optical Microscope (BX53, OLYMPUS, Japan). The root samples were cut approximately 2 mm from the root crown and observed using Confocal Laser Scanning Microscopy (CLSM, Leica Model TCS SP2, Heidelberg, Germany). The colonization of NJAU4742 strain on the cucumber root was observed with an Environmental Scanning Electron Microscope (ESEM) (XL-30, Philips, Netherlands), and CLSM (Leica Model TCS SP2, Heidelberg, Germany) with excitation wavelengths of 488 nm, and emitted light ranging from 500–600 nm collected for GFP visualization. Images were obtained using Leica confocal software (version 2.61).

Root samples (2 mm) collected from the root tip were treated by the different functional region deletion proteins (FRDP) (*Tg*SWO, ΔCBD, and ΔYoaJ) for 48 h, and then resin embedding technology was used to observed the root cell structure [71]. The sections were then examined with the Transmission Electron Microscope (Morgagni 268 Model, Philips, Netherland) operated at 80 kV. For each treatment, an average of four samples from four different roots were investigated, and 10 to 15 ultrathin sections were examined for each sample.

### Statistical analysis

Data collected included the means of the three replicates, and the statistical analysis was performed using SPSS 13.0 (SPSS Inc., Chicago, IL, USA) for calculating the mean and SE. Comparisons between groups were performed using univariate analysis in SPSS. Sequence data from this article can be found in the GenBank/NCBI data libraries.

## Supplementary information

**Additional file 1.** Additional figures and tables.

## Data Availability

The materials and datasets used and/or analyzed during the current study are available from the corresponding author on reasonable request.

## Abbreviations

FRDP: functional region deletion proteins
PGM: plant growth medium
PGPM: plant growth-promoting microbes
PDA: potato dextrose agar
CBD: carbohydrate-binding domain
ΔCBD protein: *Tg*SWO gene of lacking in the CBD domain was cloned and heterologously expressed
ΔYoaJ protein: *Tg*SWO gene of lacking in the YoaJ domain was cloned and heterologously expressed
