# Supplementary material for: TgSWO from Trichoderma guizhouense NJAU4742 promotes growth in cucumber plants by modifying the root morphology and the cell wall architecture
Source: Microb Cell Fact. 2019 Sep 3;18:148. doi: 10.1186/s12934-019-1196-8 (PMC6721366; doi:10.1186/s12934-019-1196-8)
Supplement: Supplementary file 1 — Additional file 1. Additional figures and tables. [file 12934_2019_1196_MOESM1_ESM.docx]

**Additional data for**

***Tg*SWO from *Trichoderma guizhouense* NJAU4742 promotes growth in cucumber plants by modifying the root morphology and the cell wall architecture**

Xiaohui Meng^1^, Youzhi Miao^1^, Qiumei Liu^1^, Lei Ma ^1^, Kai Guo^2^, Dongyang Liu^1^^[[1]](#footnote-1)^*, Wei Ran^1^, Qirong Shen^1^

1 Jiangsu Provincial Key Lab of Solid Organic Waste Utilization, Jiangsu Collaborative Innovation Center of Solid Organic Wastes, Educational Ministry Engineering Center of Resource-saving fertilizers, Nanjing Agricultural University, Nanjing 210095, Jiangsu, Peoples Republic of China

2 Biology Institute, Qilu University of Technology (Shandong Academy of Sciences)

**Running title:** *Tg*SWO from *Trichoderma guizhouense* NJAU4742 promotes growth in cucumber plants by modifying the root morphology and the cell wall architecture

**Email addresses:**

Xiaohui Meng: 2015203032@njau.edu.cn

Youzhi Miao: yzmiao@njau.edu.cn

Qiumei Liu: 2016203055@njau.edu.cn

Lei Ma: 2016203031@njau.edu.cn

Kai Guo: guokaicc@163.com

Wei Ran: 281633954@qq.com

Dongyang Liu: [liudongyang@njau.edu.cn](mailto:liudongyang@njau.edu.cn)

Qirong Shen: [shenqirong@njau.edu.cn](mailto:shenqirong@njau.edu.cn)

**Additional Figure Captions**

**Fig. S1 Identification of *Tgswo* knockout and overexpression mutants.** (a), the electrophoretic diagram of the KO1 and KO2 mutants; (b), the electrophoretic diagram of restriction digestion of genomic DNA of KO1 and KO2 mutants; (c), Southern Blot detection of the KO1 and KO2 mutants; (d), the electrophoretic diagram of the OE1 and OE2 mutants; (e), the *Tgswo* expression of OE mutants by quantitative real-time RT-PCR. Total RNA was extracted from *Trichoderma* mycelium (NJAU4742, OE1, OE2) at 48 h after inoculation in SM medium. RNA extracted from *Trichoderma* mycelium of NJAU4742 was used as a control. The relative expression value was calculated by the ratio of the expression value of *swollenin* to that of NJAU4742 gene *Tef1* (GenBank Accession No. Z23012.1). These results were representative of three independent experiments. Each bar represents the mean of three biological replicates with SE. Statistically significant difference as evaluated by one-way ANOVA:*, P < 0.05; **, P < 0.01, ***, P < 0.001.

**Fig. S2 the Mass spectrometry of Swollenin protein from NJAU4742.** The *Tg*SWO was electrophoretic purity, and its molecular weight was about 67 kDa. Then a mass spectrometric identification of the protein was analyzed. The matching degree of the peptide was observed in the mass spectrum database.

**Fig. S3 CLSM observations of NJAU 4742 spore attachment in cucumber root.** (a), CK, no NJAU4742 spores attached on the root surface at 24 h. (b), *Tg*SWO, a large number of NJAU 4742 spores were attached to cucumber root surfaces at 24h. The graphics were representative of at least 15 independent roots for every treatment. After 48h treated with 15 μM Swollenin, the cucumber seedlings were inoculated for 24h by the final concentration of 10^5^ germinated spores/mL under the same conditions.

**Fig. S4 the effects of KO mutants (KO1, KO2) and OE mutants (OE1, OE2) on cucumber root.** (a), Cucumber seedlings were inoculated with NJAU4742 (WT), KO mutants (KO1, KO2) and OE mutants (OE1, OE2) for five days, respectively, and WT was used as a control. The root scanning was used to observe the root physiological indexes. A, Total root length. (b), Root tips. These results were representative of three independent experiments, and each bar represents the mean of three biological replicates with SE. Statistically significant difference as evaluated by one-way ANOVA:*, P < 0.05; **, P < 0.01, ***, P < 0.001.

**Fig. S5 the effects of *Tg*SWO on cucumber growth.** (a) Effect of *Tg*SWO on the root architecture of the cucumber. Cucumber seedlings (7-day-old) grew for 48 h with 5, 10, 15, and 20 µM *Tg*SWO, respectively, and CK without *Tg*SWO was also performed under the same conditions as mentioned above. (b) The parameters of root surface area, root tips, and total root length were analysed after being treated by *Tg*SWO at different concentrations. Each treatment had three biological replicates. Different letters above the bars indicate significant differences (P < 0.05).

**Fig. S6 the effects of various FRDP on cucumber growth.** (a) Effect of various FRDP on the root architecture various FRDP of the cucumber, and the treatments, which included *Tg*SWO (15 µM), ⊿CBD (7 µM), ⊿YoaJ (10 µM) and CK (without any proteins). Cucumber seedlings (15-day-old) grew for 48 h with various FRDP. (b) The parameters of root surface area and total root length were analysed after being treated by various FRDP. Each treatment had three biological replicates. Different letters above the bars indicate significant differences (P < 0.05).

**Fig. S7 effects of *Tg*SWO from *Trichoderma guizhouense* NJAU4742 on the growth of cucumber seedlings.** Cucumber seedlings (7-day-old) grew for five days under different conditions. CK (the PGM medium + NJAU4742); T1 (the PGM medium + 1% glass fiber +NJAU4742); T2 (the PGM medium + 1% cellulose + NJAU4742); T3 (PGM medium + cucumber seedlings); T4 (PGM medium + cucumber seedlings + 1% cellulose); T5 (PGM medium + cucumber seedlings + NJAU4742); T6 (PGM medium + cucumber seedlings + 1% cellulose + NJAU4742).

**
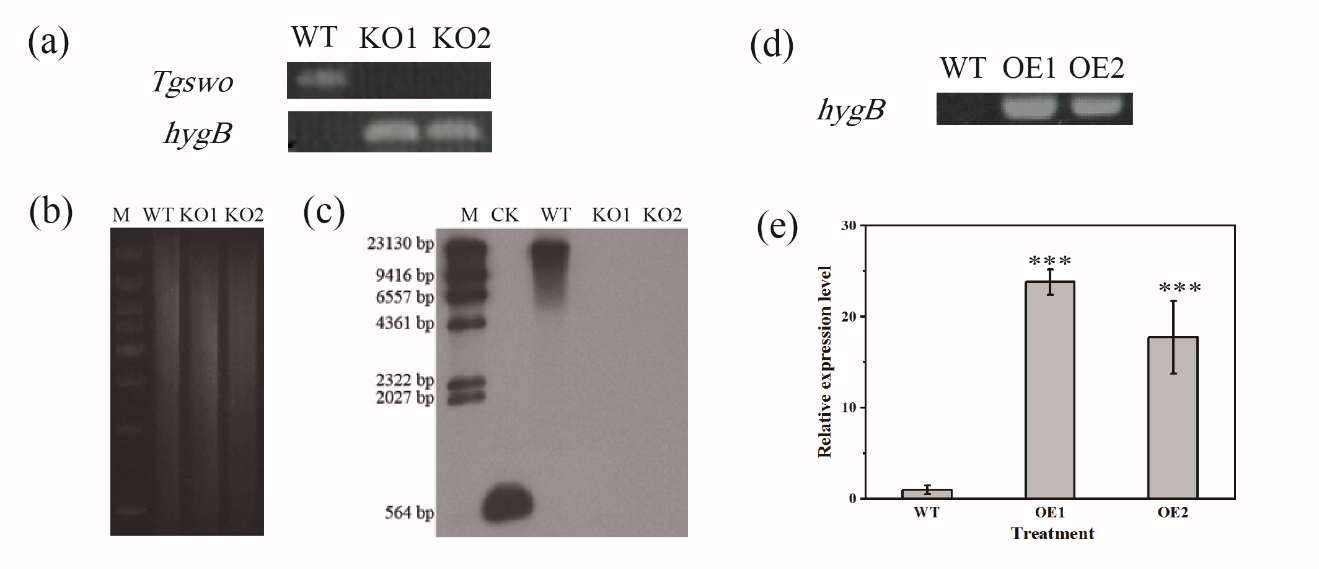
**

**Fig. S1**

**
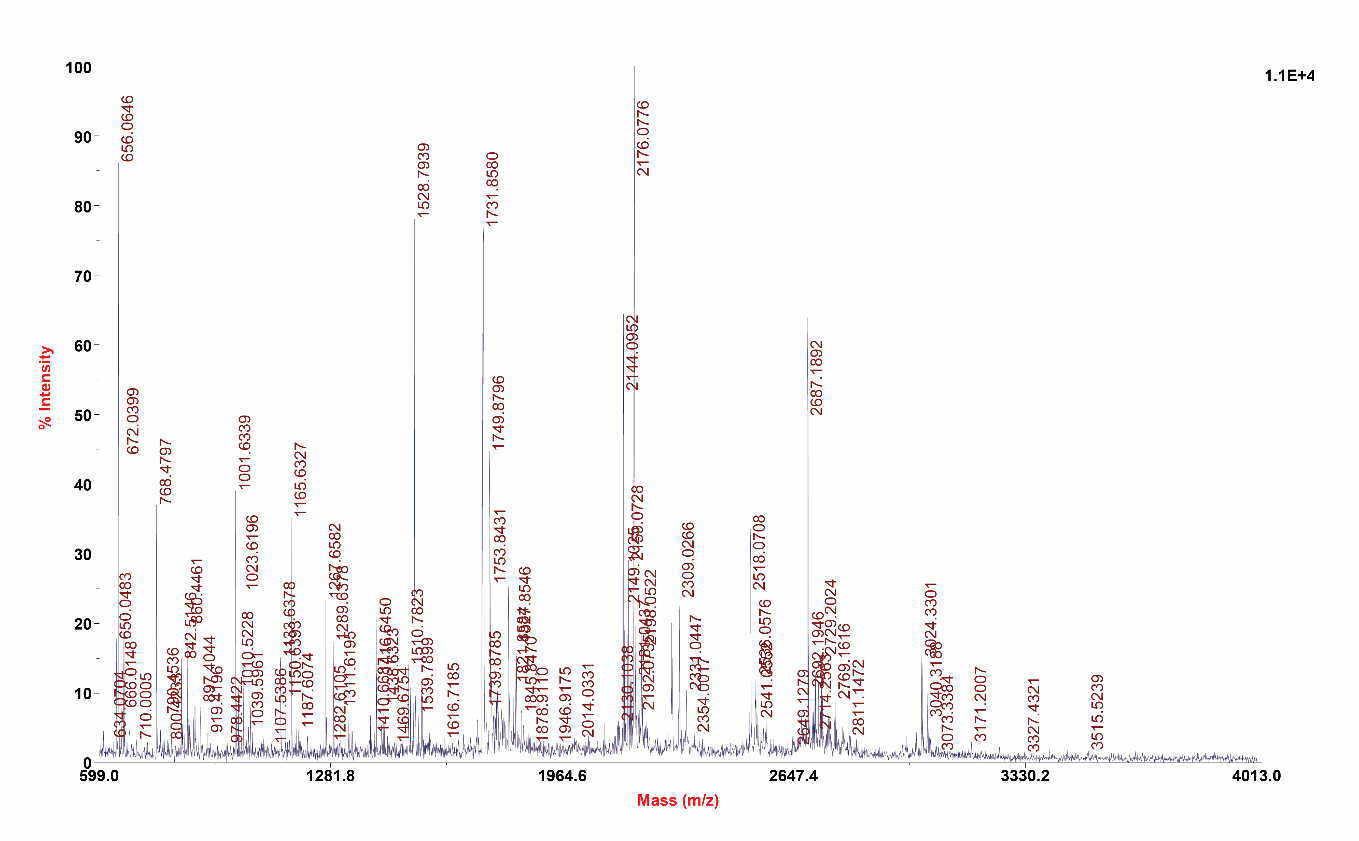
**

**Fig. S2**

**
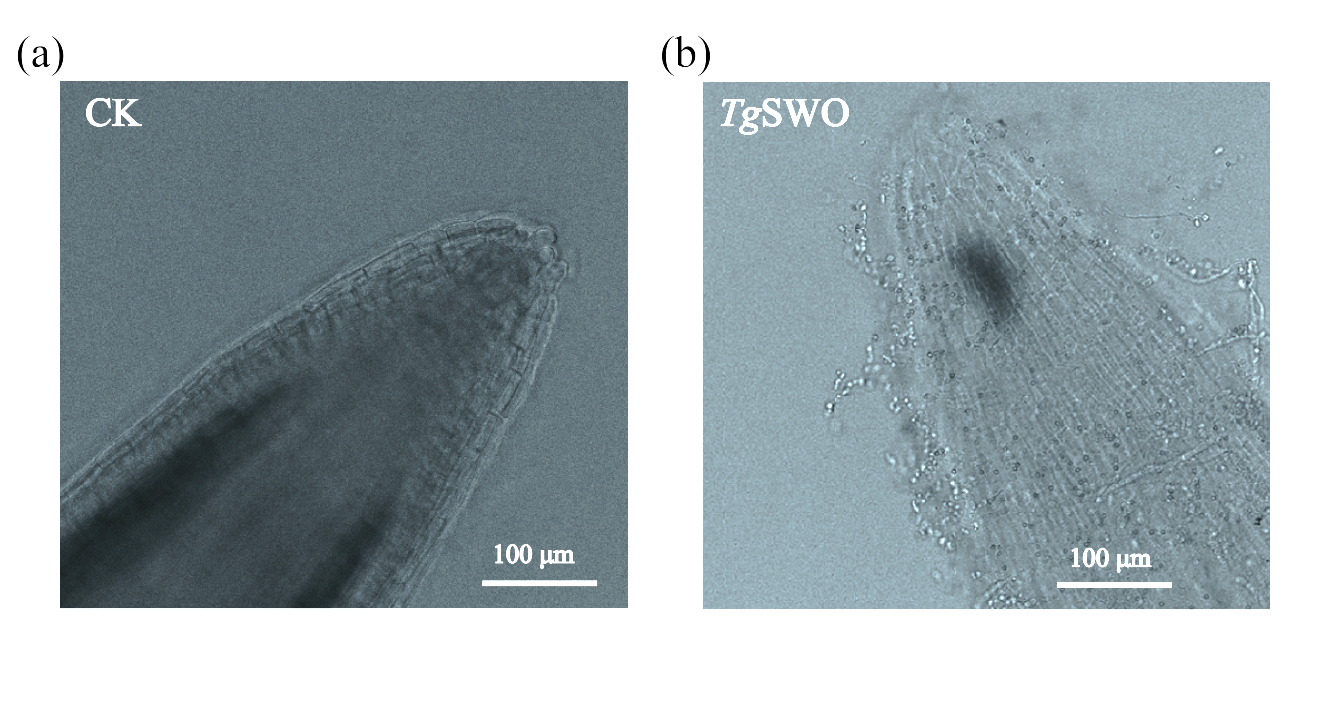
**

**Fig. S3**

**
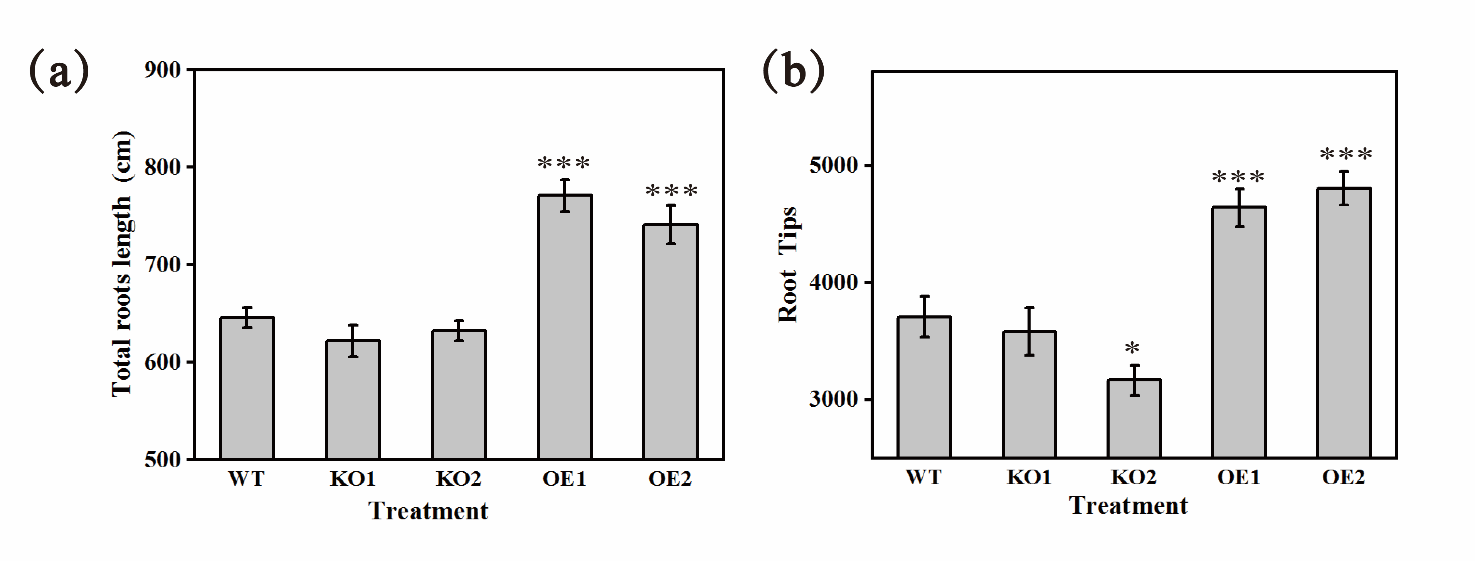
**

**Fig. S4**

**
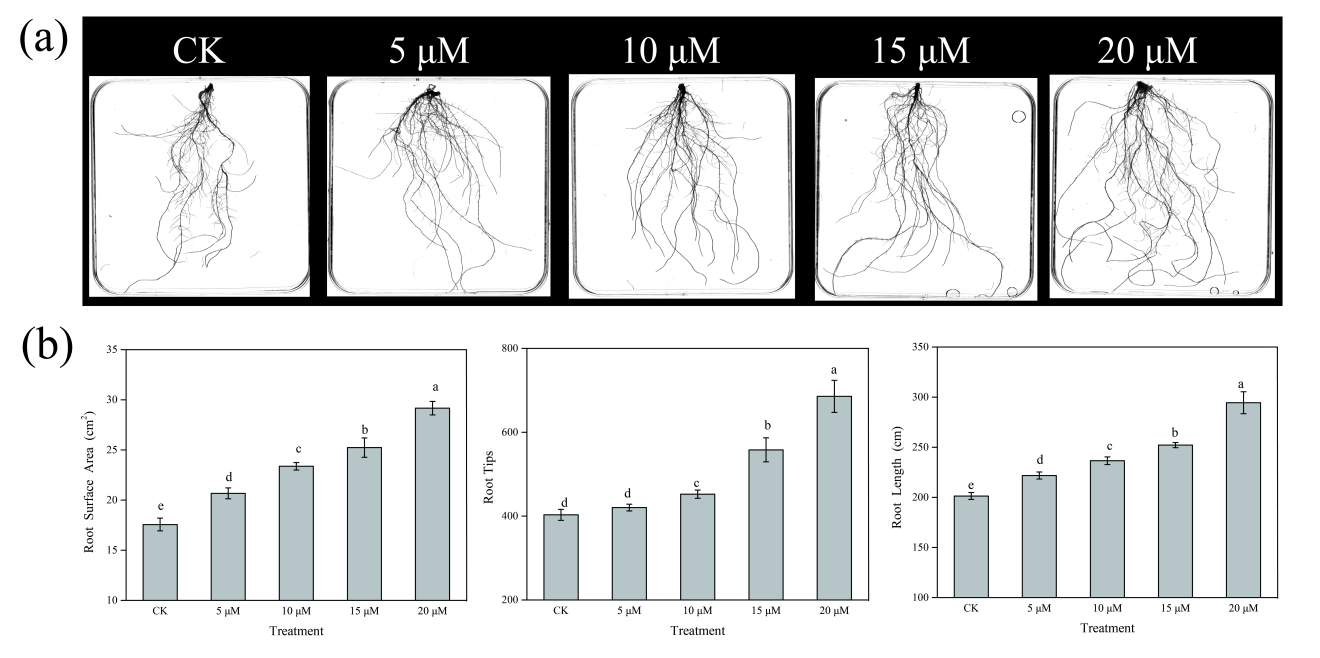
**

**Fig. S5**

**
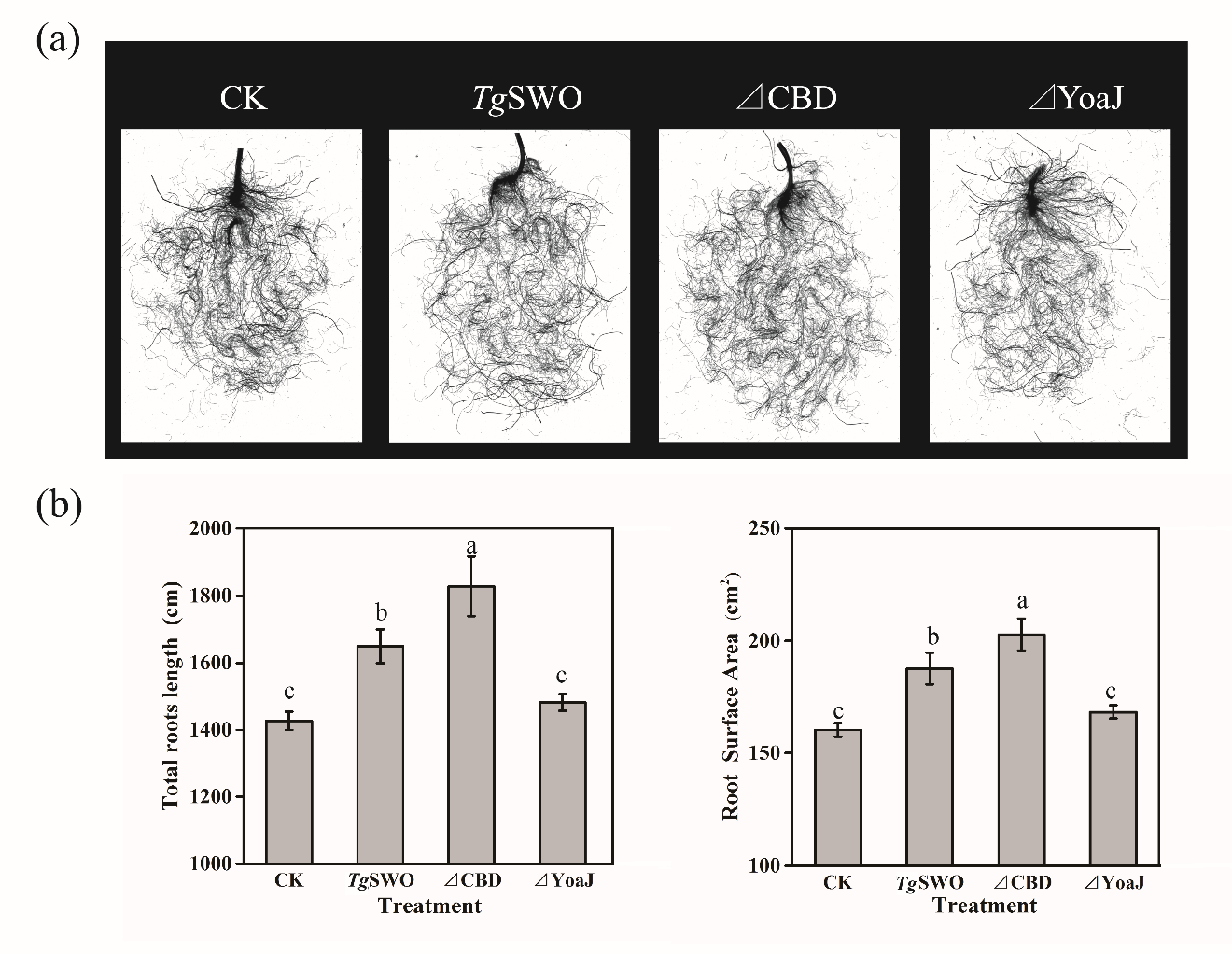
**

**Fig. S6**

**
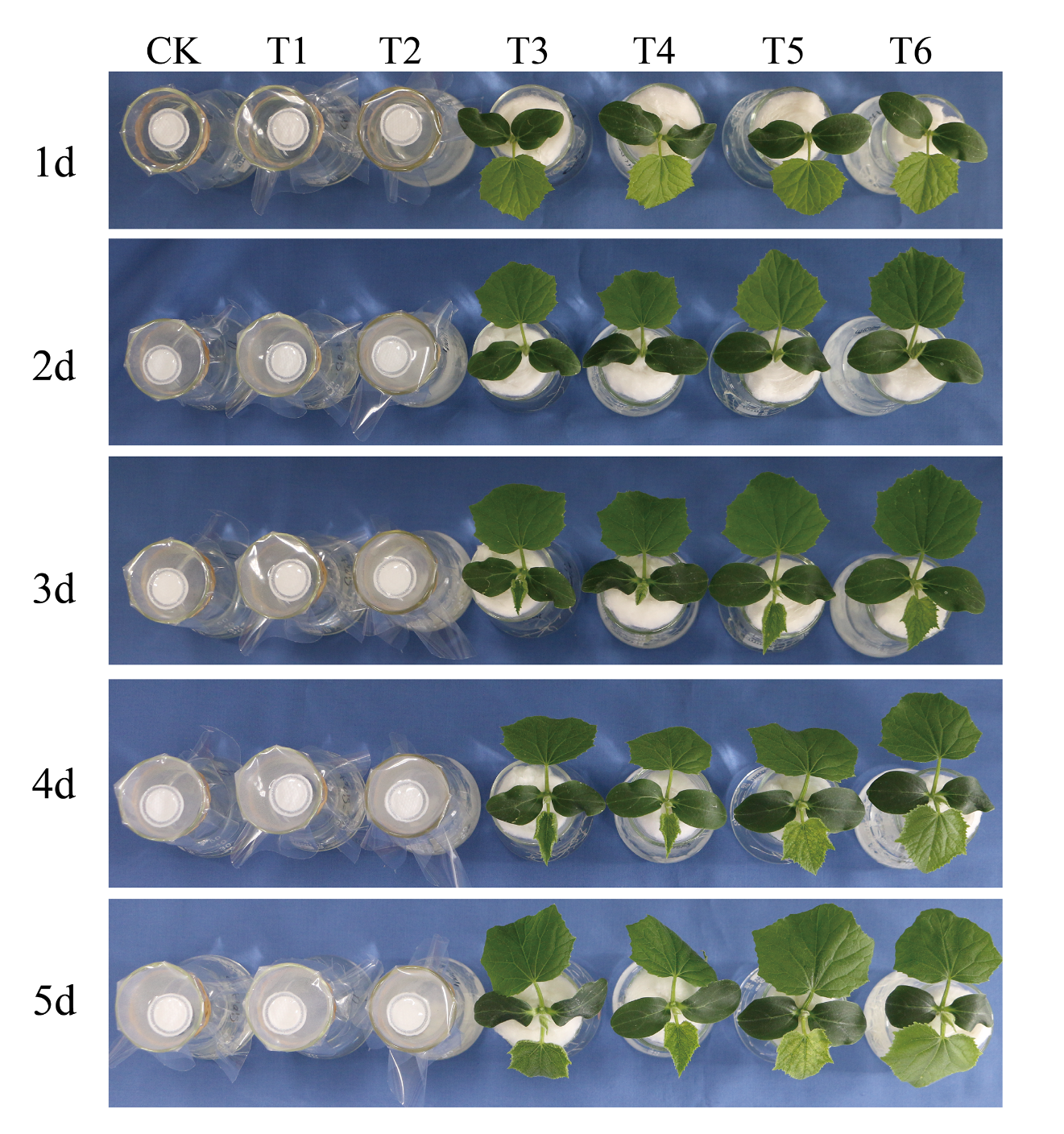
**

**Fig. S7**

| Primers | Sequence (5' - 3') |
| --- | --- |
| swo-upF | AGAGCGCGACAATCGTATCAG |
| swo-upR | GCCATATTGATGTAAGGTAGCTCTCTTCAGACGGCTTACTGACGGTC |
| swo-dF | GGGTATTCCATCTAAGCCATAGTACCATTGTCTGATCGTGCAACCCATC |
| swo-dR | CAACGACGCTTAAATACCCTTGG |
| hygB-F | GAGAGCTACCTTACATCAATATGGC |
| hygB-R | GGTACTATGGCTTAGATGGAATACCC |
| U-swoF | GCATATGATGGCAGCATTCCAG |
| D-swoR | CAAGTACAACCTAACAGCTGAGCAC |
| swoF | CAGTTTCCAGATCCTCATCTGCC |
| swoR | GCTACAAGGGCAGCTGTCTAC |
| E-swoF | CATGCCATGGCTCGTAAACTTAGTCT |
| E-swoR | GGGTTACCATAGTTTTGACTAAACTGT |
| E-hphF | TTTCCACTATCGGCGAGTACT |
| E-hphR | TGCGCCGATGGTTTCTACAAA |
| UTF | AACGTTACCAAACTGTTG |
| ITS2P | GCA GCGTTCTTCATCGAT |
| Tef1-F | TACAAGATCGGTGGTATTGGAACA |
| Tef1-R | AGCTGCTCGTGGTGCATCTC |
| F-swoF | GTGGCTTTGGCCTCTATGGT |
| F-swoR | GGTTCCGTCTGGTTTCGTCT |
| Q-swoF | CCAGACGGAACCGACTATGC |
| Q-swoR | TTGCCCTGTAAGCGACCC |
| Probe-F | AAACTTAGTCTACTGGCTCTTG |
| Probe-R | TGGTGGACTTCTGGGTAC |

Table S1 PCR primers used in this study

Table S2 the Matching of the peptide on Mass spectrometry analysis used in this study

| Query | Observed | Mr (expt) | Mr (calc) | ppm | Miss | Score | Expect | Rank | Identified Peptide |
| --- | --- | --- | --- | --- | --- | --- | --- | --- | --- |
| 5 | 860.4460 | 859.4387 | 859.4400 | -1.46 | 0 | 36 | 9.2 | 1 | DPTGTTLR |
| 20 | 1010.5261 | 1009.5188 | 1009.4862 | 32.3 | 0 | 23 | 1.1e+02 | 1 | ASSGTMITSR |
| 17 | 1001.6326 | 1000.6253 | 1000.6685 | -43.14 | 0 | 41 | 1.3 | 1 | LILLGVVFK |
| 31 | 1133.6383 | 1132.6310 | 1132.6393 | -7.30 | 0 | 52 | 0.17 | 1 | LGNVYIWLR |
| 38 | 1165.6329 | 1164.6256 | 1164.5710 | 46.9 | 0 | 34 | 11 | 1 | GVVQYGNCLR |
| 38 | 1165.6329 | 1164.6256 | 1164.5710 | 46.9 | 0 | 34 | 11 | 1 | GVVQYGNCLR |
| 44 | 1267.6584 | 1266.6511 | 1266.7118 | -47.91 | 1 | 43 | 1.3 | 1 | RPSDMPIKVPK |
| 60 | 1510.7791 | 1509.7718 | 1509.8198 | -31.78 | 1 | 35 | 8.1 | 1 | CRPGETVAVPLRR |
| 64 | 1527.7949 | 1526.7876 | 1526.8053 | -11.56 | 0 | 67 | 0.0057 | 1 | R.LQGNGSLTNGVIPTR.Y + Deamidated (NQ) |
| 77 | 1731.8584 | 1730.8511 | 1730.9301 | -45.65 | 1 | 24 | 1.2e+02 | 1 | KIVSIGSQLNNDSIIK + 3 Deamidated (NQ) |
| 113 | 2144.0940 | 2143.0867 | 2143.1215 | -16.22 | 0 | 102 | 1.8e-06 | 1 | R.YGSWVIPQGSGPFNLPVGIR.L |
| 118 | 2159.0754 | 2158.0681 | 2158.1270 | -27.27 | 1 | 21 | 2.4e+02 | 1 | YAADLTQQAIEGKIDPVIGR + Deamidated (NQ) |
| 124 | 2176.0801 | 2175.0728 | 2175.1113 | -17.69 | 0 | (59) | 0.032 | 1 | R.YGSWVIPQGSGPFNLPVGIR.L + Dioxidation (W) |
| 140 | 2309.0242 | 2308.0169 | 2308.1883 | -74.26 | 1 | 10 | 2.8e+03 | 1 | NLQASLSSGVLANRHNLTQQR + 2 Deamidated (NQ) |
| 143 | 2518.0664 | 2517.0591 | 2517.1132 | -21.47 | 0 | 57 | 0.045 | 1 | K.SGTMYPEVHHVTSNETWHYSR.S |
| 162 | 2729.2039 | 2728.1966 | 2728.2854 | -32.56 | 1 | 7 | 5.3e+03 | 1 | VNHLQDGHNALHGRFESHLDAFGR + 2 Deamidated (NQ) |
| 173 | 3024.3245 | 3023.3172 | 3023.5730 | -84.59 | 1 | 5 | 6.7e+03 | 1 | HNIRVNAIAPFFTTTHLTSGISDAWVR |

1. * To whom correspondence should be addressed: [liudongyang@njau.edu.cn](mailto:liudongyang@njau.edu.cn); Tel: +86 25 84396853; Fax: +86 25 84396853, Jiangsu Key Laboratory for Organic Solid Waste Utilization, Nanjing Agricultural University, Nanjing, CHINA. [↑](#footnote-ref-1)
